# Predicting difficult airway intubation in thyroid surgery using multiple machine learning and deep learning algorithms

**DOI:** 10.3389/fpubh.2022.937471

**Published:** 2022-08-10

**Authors:** Cheng-Mao Zhou, Ying Wang, Qiong Xue, Jian-Jun Yang, Yu Zhu

**Affiliations:** ^1^Department of Anaesthesiology, Central People's Hospital of Zhanjiang, Zhanjiang, China; ^2^Department of Anesthesiology, Pain and Perioperative Medicine, First Affiliated Hospital of Zhengzhou University, Zhengzhou, China; ^3^Anesthesia and Big Data Research Group, Central People's Hospital of Zhanjiang, Zhanjiang, China

**Keywords:** difficult airways, machine learning, deep learning, CNN, intubation

## Abstract

**Background:**

In this paper, we examine whether machine learning and deep learning can be used to predict difficult airway intubation in patients undergoing thyroid surgery.

**Methods:**

We used 10 machine learning and deep learning algorithms to establish a corresponding model through a training group, and then verify the results in a test group. We used R for the statistical analysis and constructed the machine learning prediction model in Python.

**Results:**

The top 5 weighting factors for difficult airways identified by the average algorithm in machine learning were age, sex, weight, height, and BMI. In the training group, the AUC values and accuracy and the Gradient Boosting precision were 0.932, 0.929, and 100%, respectively. As for the modeled effects of predicting difficult airways in test groups, among the models constructed by the 10 algorithms, the three algorithms with the highest AUC values were Gradient Boosting, CNN, and LGBM, with values of 0.848, 0.836, and 0.812, respectively; In addition, among the algorithms, Gradient Boosting had the highest accuracy with a value of 0.913; Additionally, among the algorithms, the Gradient Boosting algorithm had the highest precision with a value of 100%.

**Conclusion:**

According to our results, Gradient Boosting performed best overall, with an AUC >0.8, an accuracy >90%, and a precision of 100%. Besides, the top 5 weighting factors identified by the average algorithm in machine learning for difficult airways were age, sex, weight, height, and BMI.

## Introduction

Thyroid surgery is a common procedure in head and neck surgery. It often entails general anesthesia. The incidence of difficult airways during tracheal intubation (DTI) is around 10% (1). Due to the thyroid's close anatomical relationship with the larynx, laryngopharynx, and trachea, the airway may be obstructed during surgery in the presence of a large or invasive mass. This elevates the risk of anesthesia-related death and morbidity. By assessing patients' airway anatomy and pathologic changes prior to surgery, anesthesiologists can ensure safe airway management for these patients.

Presently, many intubation difficulties in thyroid surgery patients cannot be predicted in advance, due to limited predictive tools (2, 3). Machine learning has been applied to several medical fields, including cancer, pulmonary complications, chronic pain, and mental health (4–7). A study of patients with obesity has demonstrated that machine learning can help predict difficult intubations: Among the six machine learning algorithms, only three can predict intubation difficulty in patients with obesity, and the Xgbc algorithm has the best comprehensive performance, with an accuracy rate exceeding 80% (8). At present, no corresponding model has been specifically established for difficult airways in patients with thyroid problems. Among the existing models, the predictive performance is insufficient to meet clinical needs. Therefore, in this paper, we explore whether machine learning and deep learning could be used to predict difficult airways in patients undergoing thyroid surgery. In this study, we used a variety of artificial intelligence algorithms and divided the dataset into a training group and a test group. The 500 patients were randomly split into training (*N* = 350) and test (*N* = 150) cohorts. After we trained the prediction model, we verified it in the test group.

## Methods

### Ethics

The study program was approved by the Clinical Research Ethics Committee at the First Affiliated Hospital of Zhengzhou University (2021-KY-673). As the retrospective analysis was based on BioStudies' publicly accessible data, the ethics committee exempted informed consent.

### Dataset

A total of 500 patients who had undergone thyroid surgery were enrolled in this study, and difficult airway intubation occurred in 48 of them. The basic information about the patient is shown in Table 1.

**Table 1:** Basic information of patients.

**DTI**	**No**	**Yes**
*N*	452	48
Age (y)	52.88 ± 14.71	55.42 ± 11.86
Weight (kg)	69.42 ± 13.32	76.73 ± 13.16
Height (m)	166.74 ± 8.25	167.19 ± 8.52
GOITER.CIRC	37.13 ± 4.93	40.66 ± 5.29
**Sex**		
Male	100 (22.12%)	14 (29.17%)
Female	352 (77.88%)	34 (70.83%)
**BMI ≥30 kg/m^2^**		
No	397 (87.83%)	35 (72.92%)
Yes	55 (12.17%)	13 (27.08%)
**PAT**		
No	334 (73.89%)	33 (68.75%)
Yes	118 (26.11%)	15 (31.25%)
**AP.MOUTH**		
No	414 (91.59%)	35 (72.92%)
Yes	38 (8.41%)	13 (27.08%)
**MALLAMP**		
No	405 (89.60%)	30 (62.50%)
Yes	47 (10.40%)	18 (37.50%)
**NECK.MOV**		
No	368 (81.42%)	34 (70.83%)
Yes	84 (18.58%)	14 (29.17%)
**PROGNAT**		
No	422 (93.36%)	45 (93.75%)
Yes	30 (6.64%)	3 (6.25%)
**PAST.DI**		
No	450 (99.56%)	44 (91.67%)
Yes	2 (0.44%)	4 (8.33%)
**GOITER.MED**		
No	408 (90.27%)	35 (72.92%)
Yes	44 (9.73%)	13 (27.08%)
**TRACH.DEV.RX**		
No	361 (79.87%)	35 (72.92%)
Yes	91 (20.13%)	13 (27.08%)
**TMD**		
No	359 (79.42%)	30 (62.50%)
Yes	93 (20.58%)	18 (37.50%)
**NC.TMD**		
No	187 (41.37%)	9 (18.75%)
Yes	265 (58.63%)	39 (81.25%)
**EL.GANZURI**		
No	417 (92.26%)	35 (72.92%)
Yes	35 (7.74%)	13 (27.08%)

GOITER.CIRC-neck circumference (cm); PAT-Malignancy at HP; AP.MOUTH-Mouth opening <4 cm; MALLAMP-Mallampati score ≥III; NECK.MOV-Neck movement ≤ 90°; PROGNAT-Inability to prognath; PAST.DI-Past difficult intubation; GOITER.MED-Mediastinal goiter; TRACH.DEV.RX-Tracheal deviation at CXR; TMD-TMD < =6.5; NC.TMD-NC/TMD ≥5; EL.GANZURI-el-Ganzouri score ≥4.

Study participants were excluded if they had one or more anatomical abnormalities, pathology, non-standard approach, or optical fiber sober intubation, as suggested by previous procedures. DTI was defined as operations performed with correct head position and external laryngeal operation, resulting in the following: (a) difficult laryngoscopy; (b) multiple intubation attempts; (c) ineffective standard equipment and/or procedures; and (d) withdrawal and procedure reprogramming (9).

### Machine learning and deep learning methods

In this study, we used 10 algorithms, both machine learning and deep learning, to establish a corresponding model through the training group, and then verified the results in the test group.

We constructed the machine learning and deep learning models primarily in the Python language. We trained the machine learning and deep learning models, including Logistic Regression, Random Forest, Gradient Boosting, extreme gradient boosting-XGB, light gradient boosting machine-LGBM, Multilayer Perceptron Classifier-MLPC, Gaussian naïve Bayes-gnb, Convolutional Neural Network-CNN, Long Short-Term Memory- LSTM, and CNNLSTM after selecting variables for DTI prediction in the training set. Firstly, the independent variables were standardized in terms of feature ranges. We standardized our data using the sklearn library's StandardScaler software package. During the training process, we used 5-fold cross-validation to prevent model overfitting. In short, we divided the training data into 5 hierarchical subsets. Then, we trained the models using 4 subsets and validated them using the remaining subsets. In addition, we manually trained the parameters in each model. To assess the features' significance for model development, we used XGB, LGBM, and GBDT. The area under the ROC curve (AUC), accuracy, recall, precision, and F1 score served as metrics to evaluate the models. An effective model needs to produce ideal values, in both the training and the test groups. The closer the ROC curve is to the upper-left corner, the more representative the model is, that is, the AUC is close to 1. The relevant parameters of the 10 models in the training group are shown in Supplementary Table 5.

We compared the general patient data of the training group and the test group using R software. Normally distributed measurement data were expressed as x ± s, with an independent samples *t*-test used for comparison between groups; non-normally distributed measurement data were expressed as median and quartile range, with a Mann-Whitney U test used for comparison between groups. The count data were expressed as cases or percentages, with the χ2 test or Fisher's exact probability test used for comparison between groups. The inspection level α = 0.05, and we considered any difference statistically significant if *p* < 0.05.

## Results

**Figure 1:**
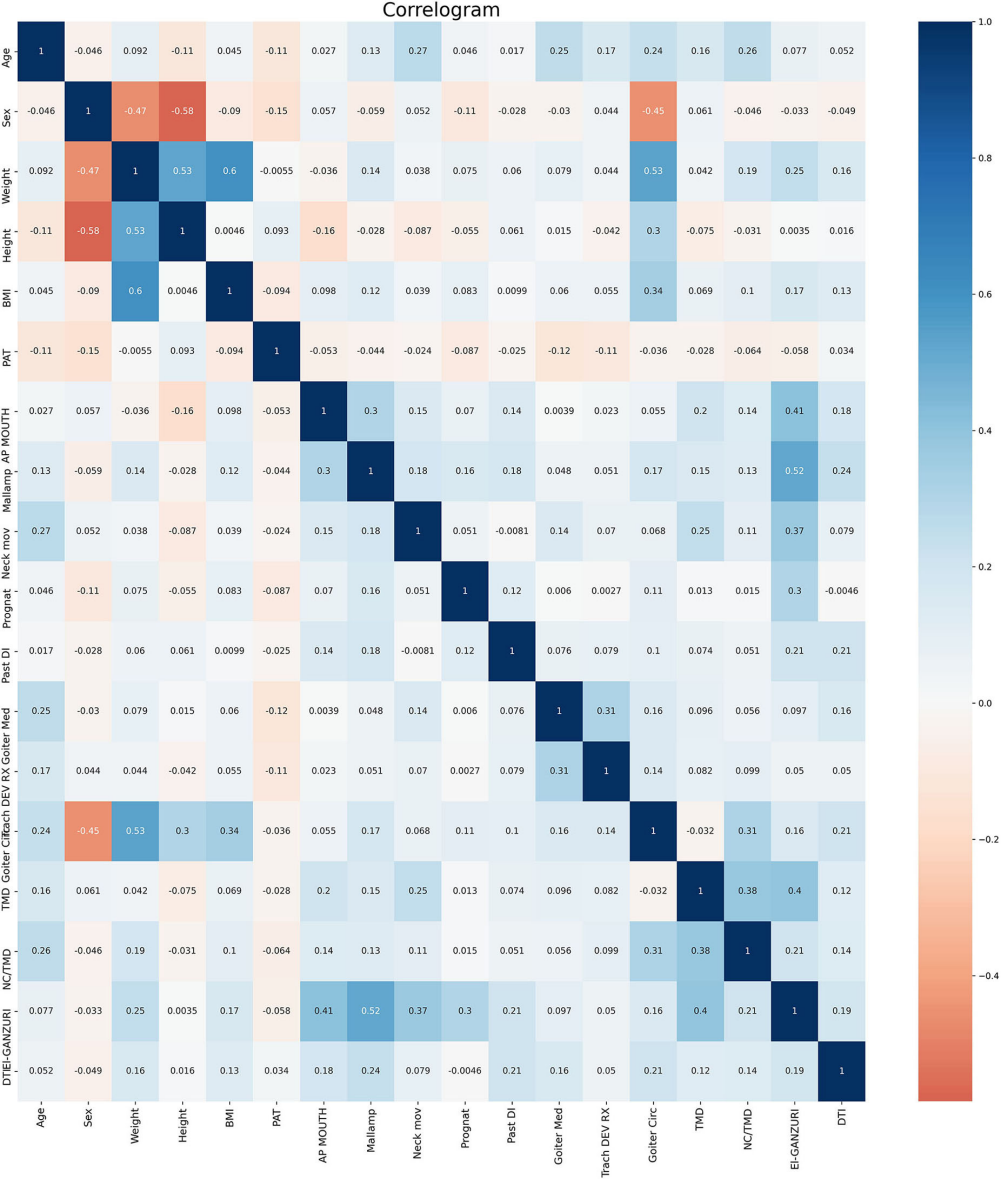
Correlation between individual variables and DIT. GOITER.CIRC-neck circumference (cm); PAT-Malignancy at HP; AP.MOUTH-Mouth opening <4 cm; MALLAMP-Mallampati score ≥III; NECK.MOV-Neck movement ≤90°; PROGNAT-Inability to prognath; PAST.DI-Past difficult intubation; GOITER.MED-Mediastinal goiter; TRACH.DEV.RX-Tracheal deviation at CXR; TMD-TMD ≤6.5; NC.TMD-NC/TMD ≥5; EL.GANZURI-el-Ganzouri score ≥4.

**Figure 2:**
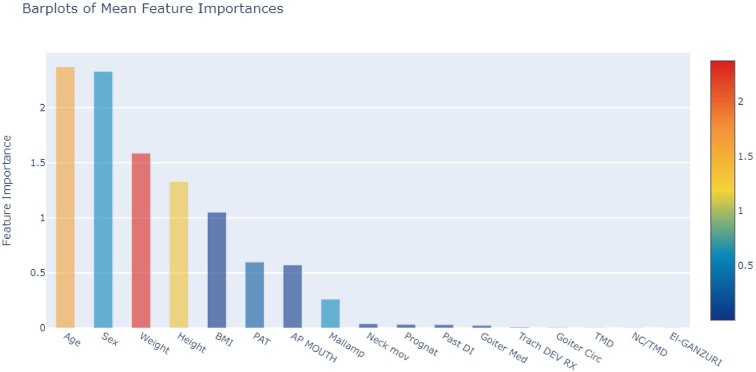
Weight analysis of individual variables in DIT (the mean machine learning algorithm).

The correlation between each variable and difficult airway could be determined via Heat Map (Figure 1). In addition, the top 5 weighting factors for difficult airways identified by the average algorithm in machine learning were age, sex, weight, height, and BMI (Figure 2). The most important influencing factor in the single GBDT and LGBM algorithms was neck circumference, while the most important influencing factor in the single XGB algorithm was sex (Supplementary Figures 1–3).

The results of the model predictions for the difficult airway in the training group are shown in Supplementary Table 2 and Supplementary Figure 4. In the training group, the AUC values and accuracy and Gradient Boosting precision were 0.932, 0.929, and 100%, respectively.

**Figure 3:**
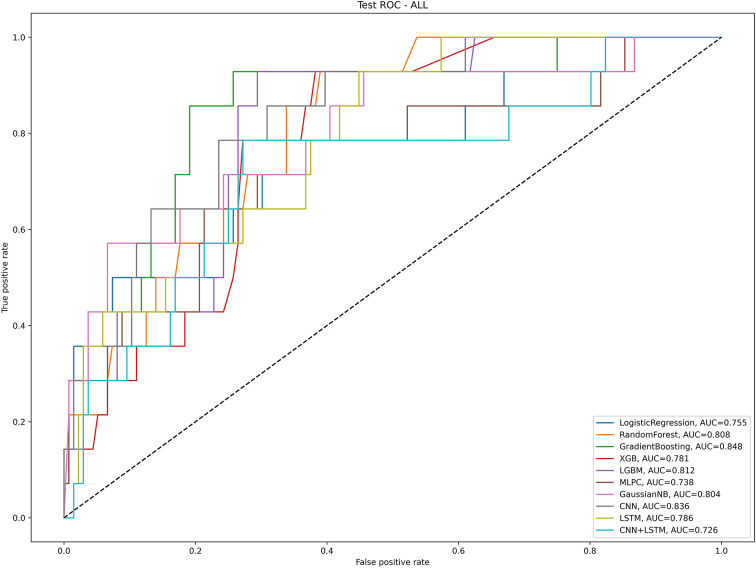
The artificial intelligence algorithm predicts the AUC value of DIT in the test group. Logistic Regression, Random Forest, Gradient Boosting, extreme gradient boosting-XGB, light gradient boosting machine-LGBM, Multilayer Perceptron Classifier-MLPC, Gaussian naive Bayes-gnb, Convolutional Neural Network-CNN, Long Short-Term Memory- LSTM and CNNLSTM.

Model effects for predicting difficult airways in the test groups: Among the models constructed by the 10 algorithms, the three algorithms with the highest AUC values were Gradient Boosting, CNN, and LGBM; their values were 0.848, 0.836, and 0.812, respectively. In addition, compared with other algorithms, the Gradient Boosting algorithm has the highest accuracy with a value of 0.913. In addition, compared with other algorithms, the Gradient Boosting algorithm has the highest precision with a value of 100% (Table 2 and Figure 3).

**Table 2:** Artificial intelligence algorithm predicts DIT results in test groups.

**Test Model name**	**Auc**	**Accuracy**	**Precision**	**Recall**	**f1**
Logistic regression	0.755	0.92	0.667	0.286	0.4
Random forest	0.808	0.913	1	0.071	0.133
Gradient boosting	0.848	0.913	1	0.071	0.133
XGB	0.781	0.913	1	0.071	0.133
LGBM	0.812	0.913	1	0.071	0.133
MLPC	0.738	0.907	0.5	0.286	0.364
gnb	0.804	0.893	0.444	0.571	0.5
CNN	0.836	0.907	0.5	0.286	0.364
LSTM	0.786	0.893	0.25	0.071	0.111
CNNLSTM	0.726	0.9	0.444	0.286	0.348

Logistic Regression, Random Forest, Gradient Boosting, extreme gradient boosting-XGB, light gradient boosting machine-LGBM, Multilayer Perceptron Classifier-MLPC, Gaussian naive Bayes-gnb, Convolutional Neural Network-CNN, Long Short Term Memory- LSTM and CNNLSTM.

The basic information results of the training group and the test group data sets are shown in Supplementary Table 3.

## Discussion

Airway management is a major concern for anesthesiologists when the thyroid gland is intubated due to goiter caused by airway distortion. Difficult or failed intubation may lead to serious complications, such as hypoxic brain injury, and even death. In recent years, machine learning prediction tools, which often outperform traditional prediction methods, have gained widespread popularity in medicine. According to our results, Gradient Boosting can deliver satisfactory results in the training and test groups, in terms of overall performance. In addition, the top 5 weighting factors identified by the average algorithm in machine learning for difficult airways are age, sex, weight, height, and BMI. The most important influencing factor in single GBDT and LGBM algorithms is neck circumference, while the most important influencing factor in the single XGB algorithm is sex.

Age has been linked to DIT in numerous studies. For example, statistics indicate that difficult or failed intubations are most common among people aged 40–59 (10). Moreover, older or middle-aged adults may struggle more with tracheal intubation than younger adults, and the predictors vary across age groups (10). Studies have shown that age- and height-based formulas can identify difficult airways early on in pediatric patients (11). However, other studies have also shown no statistically significant correlation between age and difficult intubation (12). Our findings suggest a strong correlation between age and DIT.

Many studies have shown a strong correlation between sex and DIT; the incidence of difficult tracheal intubation is higher in men than in women (*p* < 0.001) (13). Risk factors for difficult tracheal intubation include being male (14). Most likely, this is because males are the ones who increase the distance of all morphometric measurements (15). Our results also support this contention.

Moreover, height, weight, and BMI each has strong correlations with DIT. It has been shown that the ratio of height to thymic distance, and the ratio of height to sternum distance, can serve as predictors of airway difficulties (16). Moreover, the former can be used to predict tracheal intubation difficulties (17). Additionally, BMI may be a predictor of tracheal intubation difficulties in patients with obstructive sleep apnea syndrome (18). Our study also supports this contention.

There is also a strong correlation between neck circumference and DIT. In obstetric patients, a neck circumference of ≥33.5 cm is a sensitive predictor of difficult intubation (19). Thus, a neck circumference examination may help detect adverse perioperative respiratory events in children (20). In predicting intubation difficulties in the Indian population, the NC/TM ratio and Mallampatti score had better diagnostic accuracy than other bedside tests (21). Our study also supports this contention.

Many studies have also shown that machine learning and deep learning play an important role in related research in the medical field. For example, studies have shown that convolutional neural networks can be used to detect and classify COVID-19 from x-ray images (9). Studies have also shown that the path of the COVID-19 epidemic can be predicted by current evidence using machine learning algorithms (22). Studies have also shown that the use of new machine learning methods can detect Corona Virus Disease (23). Other studies have explored difficult airways and artificial intelligence. For example, studies have shown that modern machine learning methods can be used to predict difficult airways in the E.R. (24); Studies have also shown that difficult airways can be distinguished from frontal images using depth learning model sets (25). Likewise, it has been shown that the CNN algorithm can classify difficult airways (26). Our study is the first to use a large number of machine learning algorithms and deep learning algorithms simultaneously to predict difficult airways in patients with thyroid problems. We concluded that Gradient Boosting is the algorithm with the optimal comprehensive performance.

There are several limitations to this study. First, due to the retrospective nature of the data, we were unable to include new variables, such as patients' facial images, and some of their genetic transcriptomic information. This may have contributed to the model's performance. Second, we only performed internal validation of this model, and a multicenter prospective cohort validation is needed in the future. Third, for imbalanced data classification, we used f1-score and ROC-AUC curves together with the accuracy rate to evaluate the model. Fourth, feature extraction and screening would also have been of great help to the study (27), as our research entailed database data analysis, and we were unable to extract new features for research. Finally, in this study, we randomly divided the dataset into a training group and a test group. The functions of these two sample sets are as follows: the training set is used to train the supervised model, the fit model, adjust parameters, and make other choices to the algorithm; The test set is used to evaluate the effect of the trained model, but it does not change the parameters and effects of the model. It is generally used to verify whether the model is over-fitted or under-fitted, and to decide whether to retrain the model or choose another algorithm. However, more multicenter validation studies are needed in the future.

## Conclusion

Among the algorithms, Gradient Boosting performed best overall, with an AUC >0.8, an accuracy >90%, and a precision of 100%. Therefore, Gradient Boosting may be one of the preferred algorithms for future research on airway prediction among patients with thyroid difficulty.

There will be some risks and challenges in future research on difficult airways and artificial intelligence prediction models. First, the algorithm's stability and different medical scenarios may destabilize the model prediction. This would require additional subgroup analysis. Second, for the model to be applicable, we need to use simple and high-quality data to build the model. This would require establishing a high-quality database to store data.

## Data availability statement

The original contributions presented in the study are included in the article/Supplementary material, further inquiries can be directed to the corresponding author/s.

## Ethics statement

The studies involving human participants were reviewed and approved by First Affiliated Hospital of Zhengzhou University. The Ethics Committee waived the requirement of written informed consent for participation.

## Author contributions

C-MZ, YW, QX, J-JY, and YZ contributed to the data analysis, drafting, and revision of the article. All authors gave final approval of the version to be published and agreed to be accountable for all aspects of the work.

## Conflict of interest

The authors declare that the research was conducted in the absence of any commercial or financial relationships that could be construed as a potential conflict of interest.

## Publisher's note

All claims expressed in this article are solely those of the authors and do not necessarily represent those of their affiliated organizations, or those of the publisher, the editors and the reviewers. Any product that may be evaluated in this article, or claim that may be made by its manufacturer, is not guaranteed or endorsed by the publisher.

## Supplementary material

Supplementary Figure 1 Analysis of weighting each variable to DIT using the GBDT algorithm.

Supplementary Figure 2 Analysis of weighting each variable to DIT using the LGBM algorithm.

Supplementary Figure 3 Analysis of weighting each variable to DIT using the XGB algorithm.

Supplementary Figure 4 The artificial intelligence algorithm predicts the AUC value of DIT in the training group. Logistic Regression, Random Forest, Gradient Boosting, extreme gradient boosting-XGB, light gradient boosting machine-LGBM, Multilayer Perceptron Classifier-MLPC, Gaussian naive Bayes-gnb, Convolutional Neural Network-CNN, Long Short-Term Memory- LSTM and CNNLSTM.

Supplementary Table 1 Tuning parameters in the anaconda software used for each algorithm.

Supplementary Table 2 Artificial intelligence algorithm predicts DIT results in training groups.

Supplementary Table 3 The basic information results of the training group and the test group data sets.
