# Supplementary figures and images for: Predicting difficult airway intubation in thyroid surgery using multiple machine learning and deep learning algorithms
Source: Front Public Health. 2022 Aug 10;10:937471. doi: 10.3389/fpubh.2022.937471 (PMC9399522; doi:10.3389/fpubh.2022.937471)

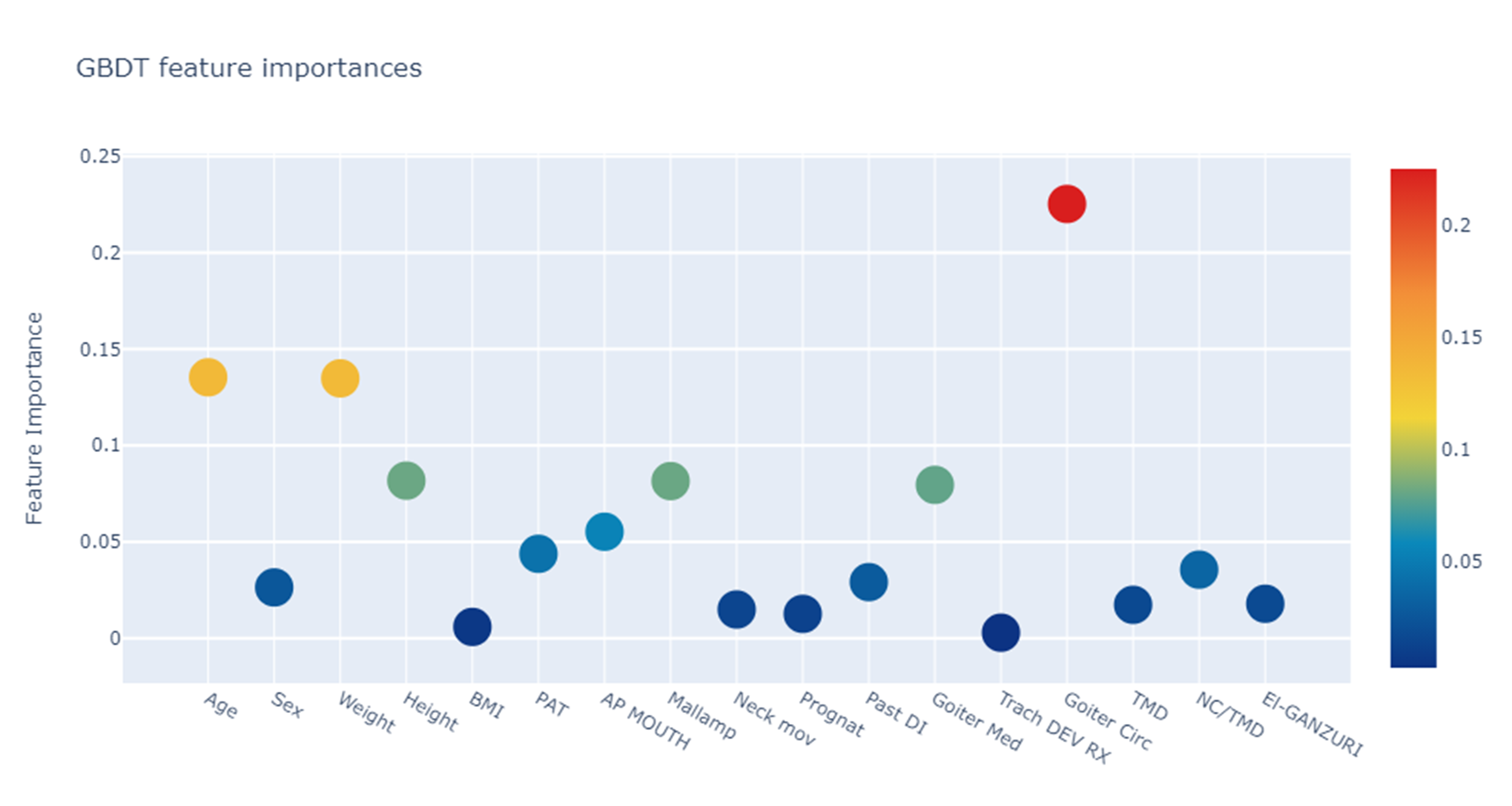

Supplement: Supplementary Figure 1 — Analysis of weighting each variable to DIT using the GBDT algorithm. [file Image_1.png]

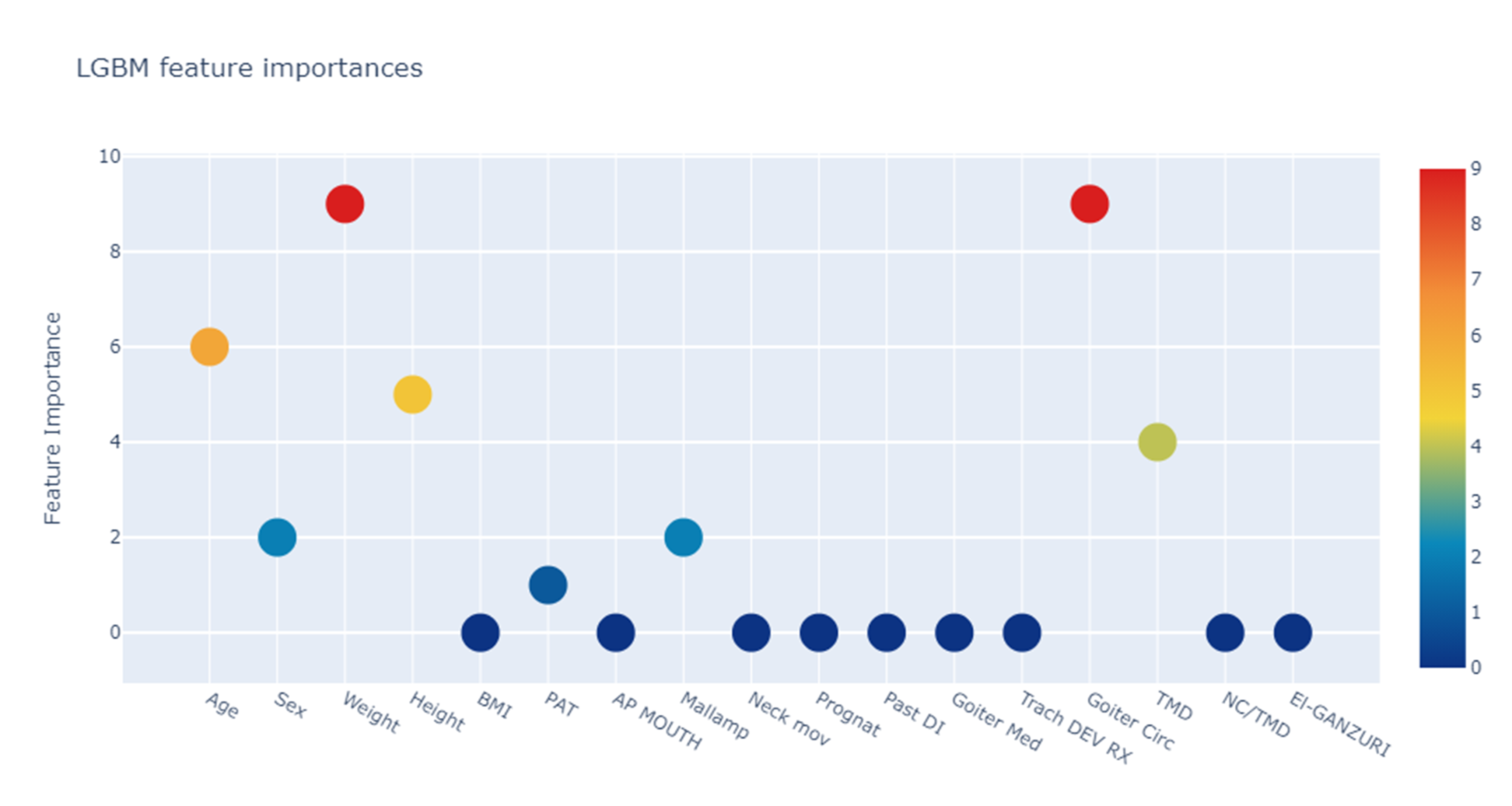

Supplement: Supplementary Figure 2 — Analysis of weighting each variable to DIT using the LGBM algorithm. [file Image_2.png]

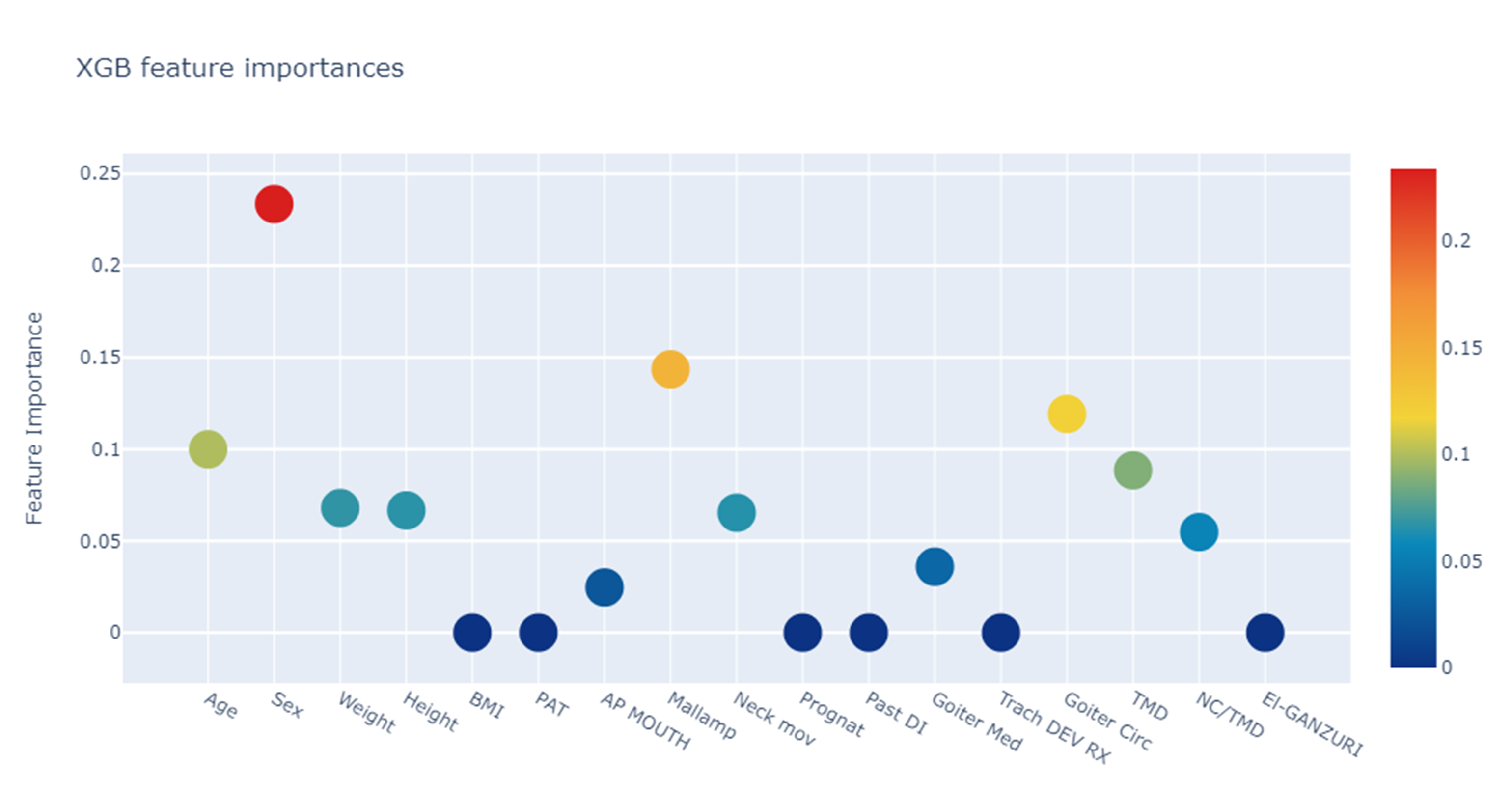

Supplement: Supplementary Figure 3 — Analysis of weighting each variable to DIT using the XGB algorithm. [file Image_3.png]
