# Supplementary material for: Predicting difficult airway intubation in thyroid surgery using multiple machine learning and deep learning algorithms
Source: Front Public Health. 2022 Aug 10;10:937471. doi: 10.3389/fpubh.2022.937471 (PMC9399522; doi:10.3389/fpubh.2022.937471)
Supplement: Supplementary Table 1 — Tuning parameters in the anaconda software used for each algorithm. [file Table_1.DOC]

Supplementary table 1. Tuning Parameters in the anaconda Software Used for Each Algorithm

| Test Model name | Tuning Parameters |
| --- | --- |
| Logistic Regression | penalty='l2',tol=0.0000001,C=10,fit_intercept=True,intercept_scaling=1,max_iter=100,multi_class='ovr',verbose=0,warm_start=False,n_jobs=1 |
| Random Forest | n_estimators=5, min_samples_leaf =5 , n_jobs = 1,random_state =1 |
| Gradient Boosting | learning_rate=0.1,n_estimators=25,max_depth=3, max_features=3,random_state =1 |
| XGB | learning_rate=0.09,n_estimators=23, max_depth=3, min_child_weight = 1,gamma=0.1,scale_pos_weight=1 |
| LGBM | learning_rate=0.09, n_estimators=26, max_depth=3 |
| MLPC | hidden_layer_sizes=30, activation='logistic', solver='adam', momentum=0.2,learning_rate_init=0.01, random_state=1 |
| gnb | - |
| CNN | model = Sequential()  model.add(Conv1D(32, 1, input_shape=(nb_features,1)))  model.add(Activation("relu"))  model.add(MaxPooling1D(2))  model.add(Conv1D(64, 1))  model.add(Activation("relu"))  model.add(MaxPooling1D(2))  model.add(Conv1D(64, 1))  model.add((Flatten()))  model.add(Dense(1, activation="sigmoid"))  model.summary() |
| LSTM | model = Sequential()  model.add(LSTM(128,input_shape=(nb_features,1)))  model.add(Dense(64))  model.add(Dense(1, activation="sigmoid"))  model.summary() |
| CNNLSTM | model = Sequential()  model.add(Conv1D(32, 1, input_shape=(nb_features,1)))  model.add(Activation("relu"))  model.add(MaxPooling1D(2))  model.add(Conv1D(64, 1))  model.add(Activation("relu"))  model.add(MaxPooling1D(2))  model.add(Conv1D(64, 1))  model.add(LSTM(128,input_shape=(nb_features,1)))  model.add(Dense(64))  model.add((Flatten()))  model.add(Dense(1, activation="sigmoid"))  model.summary() |
