# Supplementary material for: Predicting difficult airway intubation in thyroid surgery using multiple machine learning and deep learning algorithms
Source: Front Public Health. 2022 Aug 10;10:937471. doi: 10.3389/fpubh.2022.937471 (PMC9399522; doi:10.3389/fpubh.2022.937471)
Supplement: Supplementary Table 2 — Artificial intelligence algorithm predicts DIT results in training groups. [file Table_2.DOC]

Supplementary table 2 Artificial intelligence algorithm predicts DIT results in training groups

| Training Model name | auc | accuracy | precision | recall | f1 |
| --- | --- | --- | --- | --- | --- |
| Logistic Regression | 0.782 | 0.917 | 0.857 | 0.176 | 0.293 |
| Random Forest | 0.926 | 0.906 | 0.6 | 0.088 | 0.154 |
| Gradient Boosting | 0.932 | 0.929 | 1 | 0.265 | 0.419 |
| XGB | 0.911 | 0.92 | 1 | 0.176 | 0.3 |
| LGBM | 0.936 | 0.92 | 1 | 0.176 | 0.3 |
| MLPC | 0.967 | 0.969 | 1 | 0.676 | 0.807 |
| gnb | 0.734 | 0.837 | 0.244 | 0.324 | 0.278 |
| CNN | 0.793 | 0.914 | 0.833 | 0.147 | 0.25 |
| LSTM | 0.777 | 0.911 | 1 | 0.088 | 0.162 |
| CNNLSTM | 0.877 | 0.931 | 0.857 | 0.353 | 0.5 |

Note: Logistic Regression, Random Forest, Gradient Boosting, extreme gradient boosting-XGB, light gradient boosting machine-LGBM, Multilayer Perceptron Classifier-MLPC, Gaussian naive Bayes-gnb, Convolutional Neural Network-CNN, ,Long Short Term Memory- LSTM and CNNLSTM
