# Supplementary material for: Predicting difficult airway intubation in thyroid surgery using multiple machine learning and deep learning algorithms
Source: Front Public Health. 2022 Aug 10;10:937471. doi: 10.3389/fpubh.2022.937471 (PMC9399522; doi:10.3389/fpubh.2022.937471)
Supplement: Supplementary Table 3 — The basic information results of the training group and the test group data sets. [file Table_3.DOC]

Supplementary Table 3 The basic information results of the training group and the test group data sets

| DTI | Training | | | Test | | |  |  |  |
| --- | --- | --- | --- | --- | --- | --- | --- | --- | --- |
|  | No | Yes | P-value | No | Yes | P-value |  |  |  |
| N | 316 | 34 |  | 136 | 14 |  |  |  |  |
| Age (y) | 53.7 ± 14.2 | 54.1 ± 12.1 | 0.858 | 50.9 ± 15.6 | 58.6 ± 11.0 | 0.054 |  |  |  |
| Weight (kg) | 69.8 ± 13.6 | 75.4 ± 12.2 | 0.008 | 68.6 ± 12.6 | 80.0 ± 15.2 | 0.007 |  |  |  |
| Height (m) | 166.7 ± 8.2 | 166.2 ± 8.8 | 0.939 | 166.9 ± 8.3 | 169.6 ± 7.5 | 0.241 |  |  |  |
| GOITER.CIRC | 37.2 ± 4.9 | 39.6 ± 4.6 | <0.001 | 36.9 ± 5.1 | 43.3 ± 6.0 | <0.001 |  |  |  |
| Sex |  |  | 0.653 |  |  | 0.194 |  |  |  |
| Male | 64 (20.3%) | 8 (23.5%) |  | 36 (26.5%) | 6 (42.9%) |  |  |  |  |
| Female | 252 (79.7%) | 26 (76.5%) |  | 100 (73.5%) | 8 (57.1%) |  |  |  |  |
| BMI >=30 kg/m^2^ |  |  | 0.033 |  |  | 0.045 |  |  |  |
| No | 275 (87.0%) | 25 (73.5%) |  | 122 (89.7%) | 10 (71.4%) |  |  |  |  |
| Yes | 41 (13.0%) | 9 (26.5%) |  | 14 (10.3%) | 4 (28.6%) |  |  |  |  |
| PAT |  |  | 0.604 |  |  | 0.540 |  |  |  |
| No | 236 (74.7%) | 24 (70.6%) |  | 98 (72.1%) | 9 (64.3%) |  |  |  |  |
| Yes | 80 (25.3%) | 10 (29.4%) |  | 38 (27.9%) | 5 (35.7%) |  |  |  |  |
| AP.MOUTH |  |  | 0.002 |  |  | 0.005 |  |  |  |
| No | 287 (90.8%) | 25 (73.5%) |  | 127 (93.4%) | 10 (71.4%) |  |  |  |  |
| Yes | 29 (9.2%) | 9 (26.5%) |  | 9 (6.6%) | 4 (28.6%) |  |  |  |  |
| MALLAMP |  |  | <0.001 |  |  | 0.001 |  |  |  |
| No | 280 (88.6%) | 21 (61.8%) |  | 125 (91.9%) | 9 (64.3%) |  |  |  |  |
| Yes | 36 (11.4%) | 13 (38.2%) |  | 11 (8.1%) | 5 (35.7%) |  |  |  |  |
| NECK.MOV |  |  | 0.087 |  |  | 0.533 |  |  |  |
| No | 261 (82.6%) | 24 (70.6%) |  | 107 (78.7%) | 10 (71.4%) |  |  |  |  |
| Yes | 55 (17.4%) | 10 (29.4%) |  | 29 (21.3%) | 4 (28.6%) |  |  |  |  |
| PROGNAT |  |  | 0.919 |  |  | 0.977 |  |  |  |
| No | 296 (93.7%) | 32 (94.1%) |  | 126 (92.6%) | 13 (92.9%) |  |  |  |  |
| Yes | 20 (6.3%) | 2 (5.9%) |  | 10 (7.4%) | 1 (7.1%) |  |  |  |  |
| PAST.DI |  |  | 0.185 |  |  | 0.002 |  |  |  |
| No | 315 (99.7%) | 33 (97.1%) |  | 135 (99.3%) | 11 (78.6%) |  |  |  |  |
| Yes | 1 (0.3%) | 1 (2.9%) |  | 1 (0.7%) | 3 (21.4%) |  |  |  |  |
| GOITER.MED |  |  | 0.003 |  |  | 0.035 |  |  |  |
| No | 291 (92.1%) | 26 (76.5%) |  | 117 (86.0%) | 9 (64.3%) |  |  |  |  |
| Yes | 25 (7.9%) | 8 (23.5%) |  | 19 (14.0%) | 5 (35.7%) |  |  |  |  |
| TRACH.DEV.RX |  |  | 0.621 |  |  | 0.193 |  |  |  |
| No | 253 (80.1%) | 26 (76.5%) |  | 108 (79.4%) | 9 (64.3%) |  |  |  |  |
| Yes | 63 (19.9%) | 8 (23.5%) |  | 28 (20.6%) | 5 (35.7%) |  |  |  |  |
| TMD |  |  | 0.025 |  |  | 0.144 |  |  |  |
| No | 249 (78.8%) | 21 (61.8%) |  | 110 (80.9%) | 9 (64.3%) |  |  |  |  |
| Yes | 67 (21.2%) | 13 (38.2%) |  | 26 (19.1%) | 5 (35.7%) |  |  |  |  |
| NC.TMD |  |  | 0.042 |  |  | 0.012 |  |  |  |
| No | 131 (41.5%) | 8 (23.5%) |  | 56 (41.2%) | 1 (7.1%) |  |  |  |  |
| Yes | 185 (58.5%) | 26 (76.5%) |  | 80 (58.8%) | 13 (92.9%) |  |  |  |  |
| EL.GANZURI |  |  | 0.030 |  |  | <0.001 |  |  |  |
| No | 288 (91.1%) | 27 (79.4%) |  | 129 (94.9%) | 8 (57.1%) |  |  |  |  |
| Yes | 28 (8.9%) | 7 (20.6%) |  | 7 (5.1%) | 6 (42.9%) |  |  |  |  |
